# Metallic ferromagnetic films with magnetic damping under 1.4 × 10^−3^

**DOI:** 10.1038/s41467-017-00332-x

**Published:** 2017-08-10

**Authors:** Aidan J. Lee, Jack T. Brangham, Yang Cheng, Shane P. White, William T. Ruane, Bryan D. Esser, David W. McComb, P. Chris Hammel, Fengyuan Yang

**Affiliations:** 1 Department of Physics, The Ohio State University, Columbus, OH 43210 USA; 2 Center for Electron Microscopy and Analysis, Department of Materials Science and Engineering, The Ohio State University, Columbus, OH 43210 USA

## Abstract

Low-damping magnetic materials have been widely used in microwave and spintronic applications because of their low energy loss and high sensitivity. While the Gilbert damping constant can reach 10^−4^ to 10^−5^ in some insulating ferromagnets, metallic ferromagnets generally have larger damping due to magnon scattering by conduction electrons. Meanwhile, low-damping metallic ferromagnets are desired for charge-based spintronic devices. Here, we report the growth of Co_25_Fe_75_ epitaxial films with excellent crystalline quality evident by the clear Laue oscillations and exceptionally narrow rocking curve in the X-ray diffraction scans as well as from scanning transmission electron microscopy. Remarkably, the Co_25_Fe_75_ epitaxial films exhibit a damping constant <1.4 × 10^−3^, which is comparable to the values for some high-quality Y_3_Fe_5_O_12_ films. This record low damping for metallic ferromagnets offers new opportunities for charge-based applications such as spin-transfer-torque-induced switching and magnetic oscillations.

## Abstract (web summary)

Owing to their conductivity, low-damping metallic ferromagnets are preferred to insulating ferromagnets in charge-based spintronic devices, but are not yet well developed. Here the authors achieve low magnetic damping in CoFe epitaxial films which is comparable to conventional insulating ferromagnetic YIG films.

## Introduction

Insulating ferromagnets (FMs) with low Gilbert damping, such as Y_3_Fe_5_O_12_ (YIG), are advantageous for certain applications^1–11^; however, they are not suitable for spintronic devices based on charge currents, which require metallic FMs^12–17^. Ultralow-damping metallic FMs are desirable for spin-transfer-torque-induced magnetic switching and dynamics in magnetic multilayers^18^ and FM/heavy-metal structures^19^ because of the lower critical current needed. Previously, Gilbert damping constants (*α*) as low as 1.9 × 10^−3^ have been reported for epitaxial films of Fe and Fe alloys^20–22^. Recently, Schoen et al.^23^ studied magnetic damping in polycrystalline Co_*x*_Fe_1−*x*_ films of various compositions and measured a minimum *α* = 2.1 × 10^−3^ for Co_25_Fe_75_^24–26^. This ultralow damping is attributed to a minimum in the electronic density of states at the Fermi energy for *x* = 25%, since the intrinsic Gilbert damping has been shown to be proportional to the density of states at the Fermi energy in Co_*x*_Fe_1−*x*_.

These results from polycrystalline films motivated the studies reported here that were guided by the expectation that the lower defect density in high-quality epitaxial films of Co_25_Fe_75_ will lead to reduced damping^22^ as compared to their polycrystalline counterparts. In this study we grow Co_25_Fe_75_ epitaxial films using off-axis sputtering on two kinds of substrates with the goal of further reducing the magnetic damping of this promising metallic FM towards an unprecedented level. X-ray diffraction (XRD) and scanning transmission electron microscopy (STEM) verify that these films are single crystal with high crystalline quality. Variable frequency FM resonance (FMR) measurements confirm that these films exhibit significantly reduced Gilbert damping—<1.4 × 10^−3^—in contrast to polycrystalline films.

## Results

### Epitaxial growth of Co_25_Fe_75_

The growths were done using ultrahigh vacuum, off-axis sputtering with a base pressure lower than 2 × 10^−10^ Torr, which has previously been used to grow high-quality epitaxial films of various metals and oxides^27–32^. A Co_25_Fe_75_ sputter target 5 cm in diameter was prepared by annealing a pressed target of a stoichiometric Co and Fe powder mixture at 600 °C under H_2_ gas flow. The Co_25_Fe_75_ target was mounted on a horizontal sputtering source. The substrate was positioned at a horizontal distance of 5.4 cm from the target and 7.5 cm below the central axis of the target with the sample surface perpendicular to the target surface, which results in an average deposition angle of 54° relative to the target normal. We first grew 10 nm polycrystalline Co_25_Fe_75_ films on Si at room temperature with a 3 nm Cu seed layer and 5 nm Cr cap, which exhibited a low damping constant similar to that previously reported^23^. The Cr, Cu, and Co_25_Fe_75_ films were grown at Ar pressures of 10, 5, and 10 mTorr using DC sputtering with growth rates of 1.7, 3.6, and 2.4 nm/min, respectively. Co_25_Fe_75_ epitaxial films of 6.8 and 34 nm thicknesses were deposited at a substrate temperature of 300 °C directly on (001)-oriented MgO and MgAl_2_O_4_ (MAO) substrates (purchased from MTI), followed by a Cr capping layer grown at room temperature. MgO is a commonly used substrate with a lattice constant of *a* = 4.212 Å, which has a 3.9% lattice mismatch with Co_25_Fe_75_ when considering the 45° rotation between the body-centered-cubic (BCC) lattice of Co_25_Fe_75_ and the face-centered-cubic (FCC) lattice of MgO. MgAl_2_O_4_ (*a* = 8.083 Å) was used because it has a much better lattice match (within 0.4%) with Co_25_Fe_75_.

### Characterization of crystalline quality

**Fig. 1:**
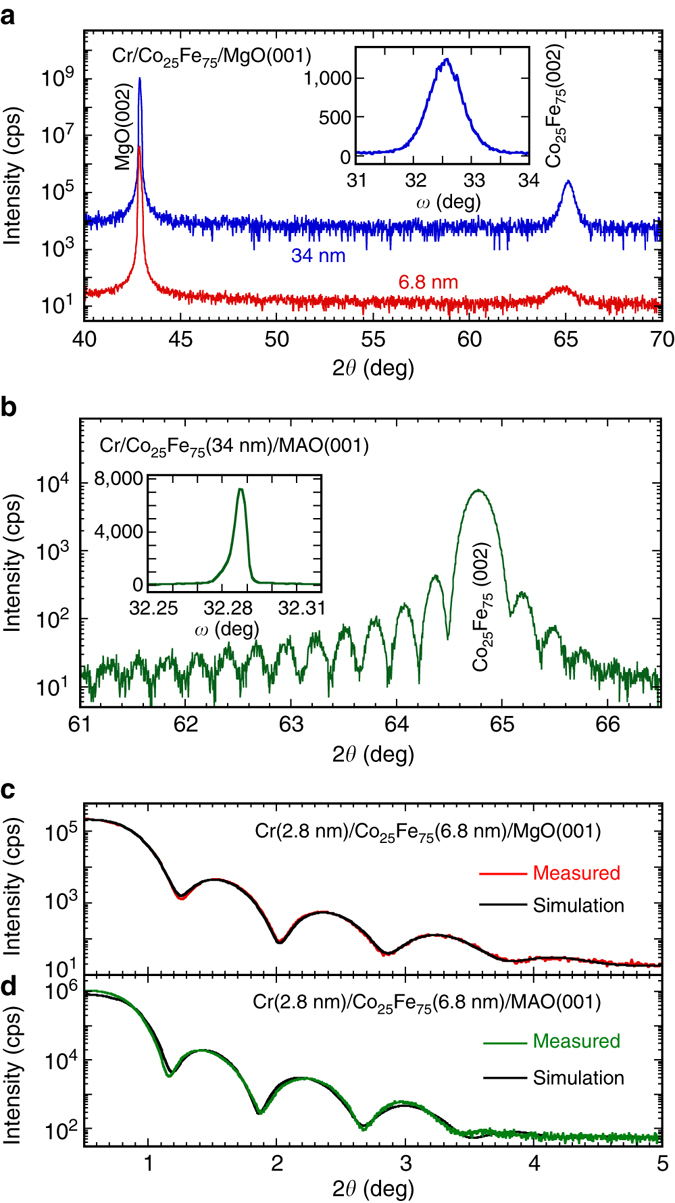
X-ray diffractometry and reflectometry of Co_25_Fe_75_ films on MgO and MgAl_2_O_4_ (MAO). **a** 2*θ*−*ω* X-ray diffraction (XRD) scans of Cr(2.8 nm)/Co_25_Fe_75_/MgO(001) films with the Co_25_Fe_75_ thickness of 6.8 and 34 nm. *Inset*: XRD rocking curve of the Co_25_Fe_75_(002) peak of the 34 nm film gives a full-width-half-maximum (FWHM) of 0.68°. **b** 2*θ*−*ω* scan of a Cr(2.8 nm)/Co_25_Fe_75_(34 nm)/MgAl_2_O_4_(001) sample. *Inset*: XRD rocking curve of the Co_25_Fe_75_(002) peak gives a FWHM of 0.0057°. X-ray reflectometry scans of **c** Cr(2.8 nm)/Co_25_Fe_75_(6.8 nm)/MgO(001) and **d** Cr(2.8 nm)/Co_25_Fe_75_(6.8 nm)/MAO(001) films, where a corresponding fit (*black*) gives a surface roughness of 0.4 nm for both samples

The crystalline quality of the epitaxial Co_25_Fe_75_ films was quantified by several methods. Figure 1a shows 2*θ*−*ω* scans obtained using triple-axis X-ray diffractometry of Cr(2.8 nm)/Co_25_Fe_75_(34 nm) and Cr(2.8 nm)/Co_25_Fe_75_(6.8 nm) films on MgO(001). The Co_25_Fe_75_(002) peaks at 65.139° and 64.844° for the 34 and 6.8 nm films correspond to out-of-plane BCC lattice constants of 2.862 and 2.873 Å, respectively. The XRD rocking curve (inset to Fig. 1a) of the Cr(2.8 nm)/Co_25_Fe_75_(34 nm)/MgO sample gives a full-width-at-half-maximum (FWHM) of 0.68°. Figure 1b shows a 2*θ*−*ω* scan of a Cr(2.8 nm)/Co_25_Fe_75_(34 nm)/MAO(001) sample, which exhibits clear Laue oscillations and a rocking curve FWHM of only 0.0057° (inset to Fig. 1b). The pronounced Laue oscillations and exceptionally narrow rocking curve are rarely seen in metallic epitaxial films, demonstrating very high crystalline quality enabled by the excellent lattice match between Co_25_Fe_75_ and MAO. Figure 1c, d show the X-ray reflectometry scans of a Cr(2.8 nm)/Co_25_Fe_75_(6.8 nm) bilayer on MgO and MAO, respectively, where the fitting gives a film roughness of 0.4 nm for both samples.

**Fig. 2:**
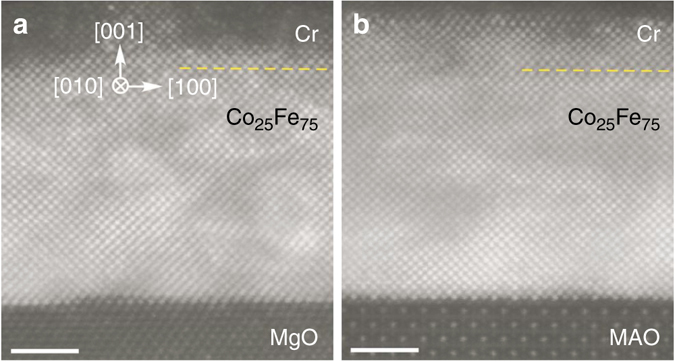
Atomic resolution images of Co_25_Fe_75_ films. Scanning transmission electron microscopy images of **a** Cr(2.8 nm)/Co_25_Fe_75_(6.8 nm)/MgO and **b** Cr(2.8 nm)/Co_25_Fe_75_(6.8 nm)/MAO films viewed along the Co_25_Fe_75_ [010] axis, where the boundary between Cr and Co_25_Fe_75_ as confirmed by electron energy loss spectroscopy scans is indicated by the dashed line. In **a**, the less clear Co_25_Fe_75_ atomic columns near the middle within 2 nm from the interface is due to strain relaxation via the disappearance of a Co_25_Fe_75_ column (MgO lattice constant is 3.9% larger than that of Co_25_Fe_75_). In **b**, the Co_25_Fe_75_ lattice matches perfectly with that of MAO without strain relaxation and its associated defects. *Scale bars*: 2 nm

**Fig. 3:**
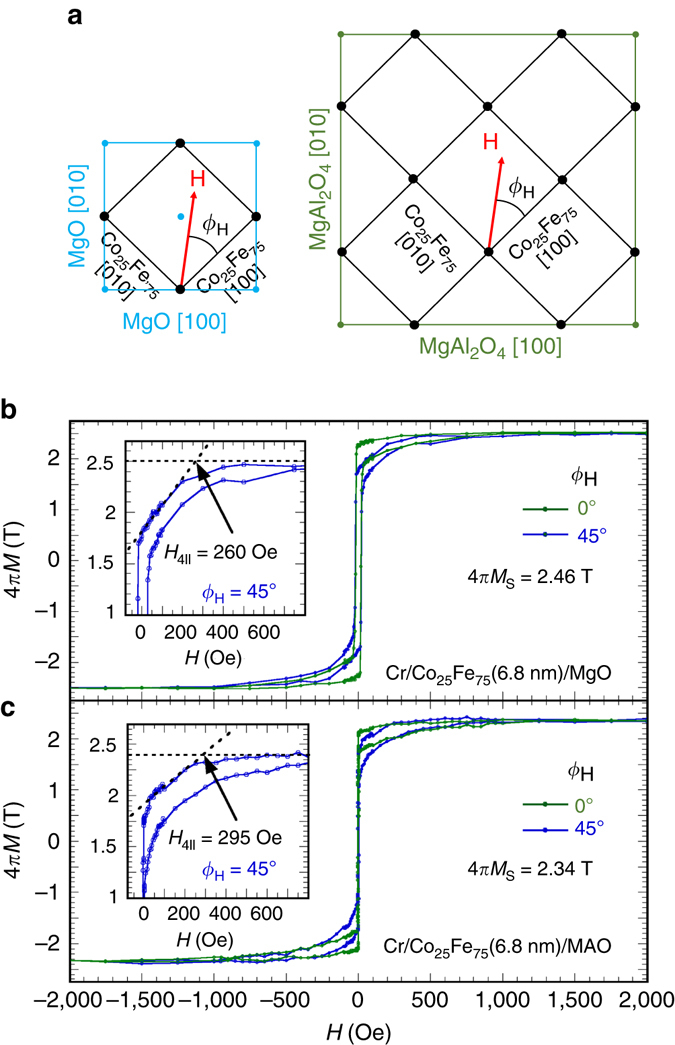
Determination of in-plane magnetic hard axis. **a** Schematic illustration of the epitaxial relationship between the Co_25_Fe_75_ lattice and the MgO and MAO(001) substrates as well as the in-plane field angle *ϕ*_H_ with respect to the Co_25_Fe_75_ [100] axis. In-plane magnetic hysteresis loops for the **b** Cr(2.8 nm)/Co_25_Fe_75_(6.8 nm)/MgO(001) and **c** Cr(2.8 nm)/Co_25_Fe_75_(6.8 nm)/MAO(001) sample for field applied along a Co_25_Fe_75_ [100] axis (*ϕ*_H_ = 0°, easy axis) and a Co_25_Fe_75_ [110] axis (*ϕ*_H_ = 45°, hard axis). A saturation magnetization of 2.46 and 2.34 T as well as an easy-axis coercive field of 20 and 2.5 Oe are obtained for the 6.8 nm Co_25_Fe_75_ film on MgO and MAO, respectively. The insets in **b** and **c** show a zoom-in view of the hard-axes hysteresis loops (*ϕ*_H_ = 45°) to extract the in-plane magnetocrystalline anisotropy, *H*_4||_ = 260 and 295 Oe, for the Co_25_Fe_75_ film on MgO and MAO, respectively

The Cr(2.8 nm)/Co_25_Fe_75_(6.8 nm) films on MgO and MAO were characterized by high-angle annular dark field scanning transmission electron microscopy (STEM) using an FEI probe-corrected Titan^3^ 80–300 S/TEM. Figure 2 shows the STEM images viewed along the Co_25_Fe_75_ [010] direction (MgO and MAO [110] axis), which reveal the BCC ordering of Co_25_Fe_75_ and its epitaxy on the substrates. Electron energy loss spectroscopy was used to determine the Cr/Co_25_Fe_75_ boundary, which is unclear in the STEM images because of the small difference in their atomic numbers. The STEM images also show the epitaxial relationship between Co_25_Fe_75_ and the substrates as illustrated in Fig. 3a, where the BCC lattice of Co_25_Fe_75_ grows at a 45° in-plane rotation relative to the FCC lattice of MgO and the spinel lattice of MAO.

### Characterization of in-plane magnetization

Magnetic hysteresis loops of Cr(2.8 nm)/Co_25_Fe_75_(6.8 nm) on MgO and MAO were measured at room temperature using a vibrating sample magnetometer at various in-plane orientations defined by *ϕ*_*H*_, the angle between the applied magnetic field (*H*) and the Co_25_Fe_75_ [100] axis (see Fig. 3a). Figure 3b shows two hysteresis loops for Cr(2.8 nm)/Co_25_Fe_75_(6.8 nm)/MgO measured at *ϕ*_*H*_ = 0° and 45° where the small diamagnetic background from MgO has been subtracted. The *ϕ*_*H*_ = 0° loop has a sharp magnetic reversal with a coercive field (*H*_c_) of 20 Oe while the *ϕ*_*H*_ = 45° loop has a higher saturation field and *H*_c_ = 23 Oe. By comparing the two hysteresis loops, we conclude that Co_25_Fe_75_ [100] (*ϕ*_*H*_ = 0°) is the easy axis and Co_25_Fe_75_ [110] (*ϕ*_*H*_ = 45°) is the in-plane hard axis within the framework of in-plane cubic anisotropy. The saturation magnetization, 4*πM*_s_ = 2.46 ± 0.02 T, of the film on MgO is extracted from the hysteresis loop, which has been confirmed by a measurement using a SQUID magnetometer.

The inset to Fig. 3b shows a closer view of the hard-axis loop (*ϕ*_*H*_ = 45°), which allows us to obtain the in-plane magnetocrystalline anisotropy. By applying separate linear fits to the saturated high field regime and the region between 200 and 0 Oe, we extract the in-plane magnetocrystalline anisotropy of 260 ± 10 Oe between the [100] and [110] axes^33^. This is in agreement with the in-plane cubic anisotropy *H*_4||_ obtained from FMR measurements described below. Figure 3c shows the in-plane hysteresis loops for Cr(2.8 nm)/Co_25_Fe_75_(6.8 nm)/MAO, from which we obtain 4*πM*_s_ = 2.34 ± 0.02 T and an in-plane magnetocrystalline anisotropy of 295 ± 10 Oe.

**Fig. 4:**
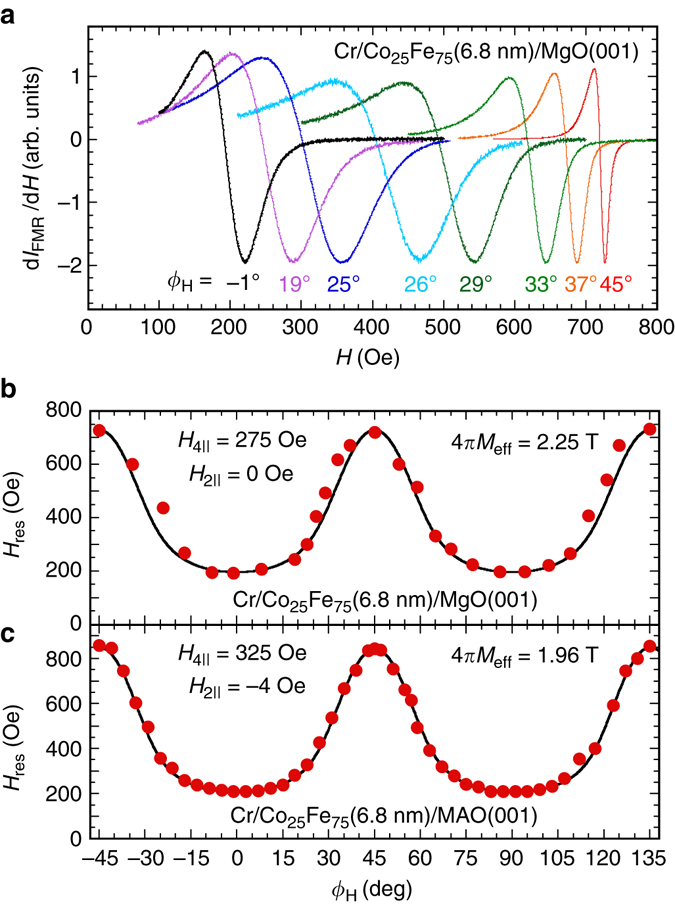
Angular dependence of ferromagnetic resonance. **a** Derivative ferromagnetic resonance (FMR) absorption spectra for a Cr(2.8 nm)/Co_25_Fe_75_(6.8 nm)/MgO(001) sample at in-plane angles *ϕ*_H_ from −1° to 45°. **b** The resonance fields of the FMR spectra in **a** plotted against *ϕ*_H_. From the fit to the experimental data the values for *H*_4||_, *H*_2||_, and 4*πM*_eff_ are extracted. **c** A similar *H*_res_ vs. *ϕ*_H_ plot for a Cr(2.8 nm)/Co_25_Fe_75_(6.8 nm)/MAO(001) sample

To probe the dynamic magnetic properties of the Co_25_Fe_75_ epitaxial films, we performed angular-dependent FMR measurements on our films. Figure 4a shows the derivative spectra of FMR absorption for the Cr(2.8 nm)/Co_25_Fe_75_(6.8 nm)/MgO sample for various *ϕ*_*H*_ from −1° to 45° taken at a microwave frequency of *f* = 9.66 GHz in a cavity, from which the resonant field (*H*_res_) is obtained. From the in-plane angular dependence of *H*_res_, we can determine the magnetic anisotropy of the Co_25_Fe_75_ film. Magnetization subject to an energy landscape with cubic symmetry can be quantitatively described by the total free energy density (*F*) given by^15, 34^,

1 $$\begin{array}{ccccc}F = - {\bf{H}} \cdot {\bf{M}} + \frac{1}{2}M\left\{ {4\pi {M_{{\rm{eff}}}}{\rm{co}}{{\rm{s}}^2}\theta - \frac{1}{2}{H_{4 \bot }}{\rm{co}}{{\rm{s}}^4}\theta } \right.\\ \\ \left. {- \frac{1}{8}{H_{4||}}\left( {3 + {\rm{co}}{{\rm{s}}^4}\phi } \right){\rm{si}}{{\rm{n}}^4}\theta - {H_{2||}}{\rm{si}}{{\rm{n}}^2}\theta {\rm{si}}{{\rm{n}}^2}\left( {\phi - \frac{\pi }{4}} \right)} \right\},\end{array}$$

where *θ* is the out-of-plane angle and *ϕ* the in-plane angle for the orientation of the equilibrium magnetization (**M**) with respect to the easy axis, 4*πM*_eff_ *= *4*πM*_s_−*H*_2⊥_ is the effective saturation magnetization, and *H*_2⊥_ is the out-of-plane uniaxial anisotropy. *H*_4⊥_, *H*_4∥_, and *H*_2∥_ are the out-of-plane cubic, in-plane cubic, and in-plane uniaxial anisotropy, respectively. By substituting Eq. (1) into the FMR resonance condition^15, 34^,

2 $${\left( {\frac{\omega }{\gamma }} \right)^{\!\!2}} = \frac{1}{{{M^2}{\rm{si}}{{\rm{n}}^2}\theta }}\left[ {\frac{{{\partial ^2}F}}{{\partial {\theta ^2}}}\frac{{{\partial ^2}F}}{{\partial {\phi ^2}}} - {{\left( {\frac{{{\partial ^2}F}}{{\partial \phi \partial \theta }}} \right)}^{\!\!2}}} \right],$$

we can calculate *H*_res_ for a given *ϕ*_*H*_, where *γ* is the gyromagnetic ratio and *ω* is the resonance angular frequency. For in-plane angular FMR, *θ* = 90° and the *H*_4⊥_ term drops out; the in-plane equilibrium angle *ϕ* is determined by numerically minimizing the free energy density.

Figure 4b shows the in-plane angular dependence of *H*_res_ for Cr(2.8 nm)/Co_25_Fe_75_(6.8 nm)/MgO and the fit using Eq. (2) and *γ*/2*π* = 29.5 ± 1.0 GHz T^−1^ ^35^, which agrees well with the experimental data and gives *H*_2∥_ = 0 ± 5 Oe, *H*_4∥_ = 275 ± 5 Oe, and 4*πM*_eff_ = 2.25 ± 0.02 T. The obtained *H*_4||_ agrees with the value (260 Oe) determined from the magnetometry measurements in Fig 3b. Figure 4c shows the in-plane angular dependence of *H*_res_ for Cr(2.8 nm)/Co_25_Fe_75_(6.8 nm)/MAO. From the fitting to the data, we obtain *H*_2∥_ = −5 ± 5 Oe, *H*_4∥_ = 325 ± 5 Oe, and 4*πM*_eff_ = 1.96 ± 0.02 T, where the value of *H*_4||_ is close to the anisotropy (295 Oe) obtained in Fig. 3c.

### Measurement of Gilbert damping

**Fig. 5:**
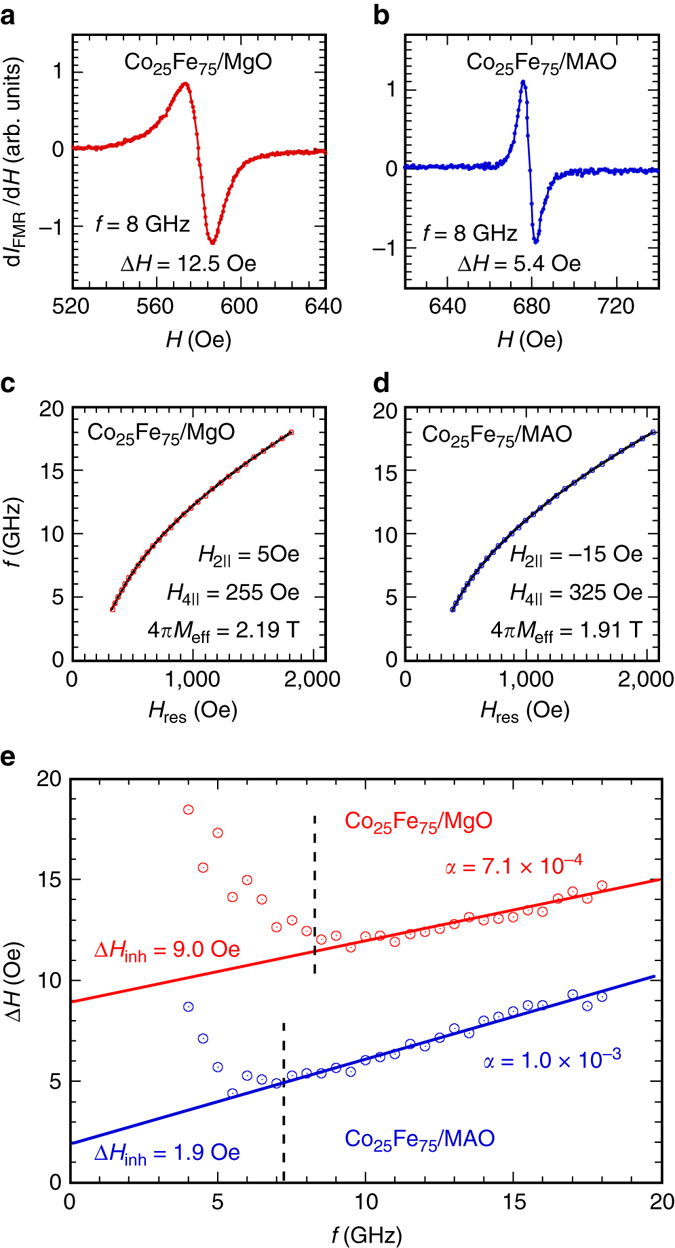
Determination of Gilbert damping. Representative ferromagnetic resonance (FMR) absorption derivative spectra for the **a** Cr(2.8 nm)/Co_25_Fe_75_(6.8 nm)/MgO(001) and **b** Cr(2.8 nm)/Co_25_Fe_75_(6.8 nm)/MAO(001) sample at *ϕ*_H_ = 45° measured at *f* = 8 GHz, which give a FMR linewidth of 12.5 and 5.4 Oe, respectively. Frequency vs. resonance field plots for the Co_25_Fe_75_ films on **c** MgO and **d** MAO measured at *ϕ*_H_ = 45°, from which the anisotropy terms and effective saturation magnetization are extracted. **e** Frequency dependencies of the FMR linewidths for the Co_25_Fe_75_ films on MgO (red) and MAO (*blue*), where the behavior below 8 and 7 GHz, respectively, reflects incomplete saturation of the films leading to increases in the inhomogeneous broadening. The *solid lines* are linear fits to the frequency range above 8 (7)  GHz for the film on MgO (MAO), from which the Gilbert damping and inhomogeneous broadening are extracted

Frequency-dependent FMR absorption was measured between 3 and 18 GHz using a microwave stripline by sweeping the magnetic field at various fixed frequencies. A small modulation of the magnetic field was applied to enable the measurement of differential absorbed power with a Schottky diode detector and a lock-in amplifier. Figure 5a, b show representative FMR spectra at *f* = 8 GHz with the magnetic field applied along the Co_25_Fe_75_ [110] axis for the Cr(2.8 nm)/Co_25_Fe_75_(6.8 nm)/MgO and Cr(2.8 nm)/Co_25_Fe_75_(6.8 nm)/MAO samples, which exhibit a peak-to-peak linewidth (*∆H*) of 12.5 and 5.4 Oe, respectively. The FMR linewidth of 5.4 Oe at 8 GHz rivals that of high-quality YIG films and is unprecedented for metallic FM films. We fit the *f* vs. *H*_res_ plots in Fig. 5c, d using the same procedure for Fig. 4. The extracted values for in-plane anisotropies and effective saturation magnetization are: *H*_4∥_ = 260 ± 5 Oe, *H*_2∥_ = 0 ± 5 Oe, and 4*πM*_eff_ = 2.19 ± 0.02 T for the film on MgO; *H*_4∥_ = 315 ± 10 Oe, *H*_2∥_ = −5 ± 5 Oe, and 4*πM*_eff_ = 1.91 ± 0.02 T for the film on MAO, which agree with the results in Fig. 4.

Figure 5e shows the corresponding frequency dependencies of *∆H* for the two samples. Below ~8 (7) GHz for the film on MgO (MAO), the data deviate from the linear dependence found above it. When comparing the hard-axis saturation fields of ~ 600 Oe to the *H*_res_ of 581 Oe (592 Oe) at 8 (7)  GHz for the film on MgO (MAO), we can understand these two regimes as follows. The films exhibit linear frequency dependence of *∆H* above a threshold field where the magnetization is fully aligned. Below the threshold, the magnetization is not fully aligned, leading to non-uniform magnetization which inhomogeneously broadens the linewidth^16^. We first apply a linear fit in Fig. 5e in the frequency regime above 8 (7) GHz for the film on MgO (MAO), from which the damping constant can be determined using Δ*H* = Δ*H*_inh_ + $\frac{{4\pi \alpha f}}{{\sqrt 3 \gamma }}$, where Δ*H*_inh_ is the inhomogeneous broadening^36^. For Co_25_Fe_75_(6.8 nm)/MgO, *α* = (7.1 ± 0.6) × 10^−4^ and Δ*H*_inh_ = 9.2 ± 0.3 Oe; for Co_25_Fe_75_(6.8 nm)/MAO, *α* = (1.16 ± 0.02) × 10^−3^ and Δ*H*_inh_ = 1.8 ± 0.1 Oe. If we choose a higher threshold field corresponding to 10 (9)  GHz for the film on MgO (MAO), we obtain *α* = (8.5 ± 0.6) × 10^−4^ and Δ*H*_inh_ = 8.4 ± 0.3 Oe for the film on MgO, and *α* = (1.21 ± 0.02) × 10^−3^ and Δ*H*_inh_ = 1.5 ± 0.1 Oe for the film on MAO. The small Δ*H*_inh_ in the Co_25_Fe_75_ film on MAO can be attributed to the high crystalline quality of the film. The damping constant in the film on MAO is slightly larger than that on MgO, for which the reason is unknown at this point. We note that FMR measurements at higher frequencies, e.g., 40 or 70 GHz, would further improve the accuracy for the extracted values of intrinsic damping in our films; however, we do not have this capability and will pursue such measurements through collaboration in the future. Based on our measurements, we are confident that the intrinsic damping constant in our epitaxial Co_25_Fe_75_ films is below 1.4 × 10^−3^, which is obtained when we assume Δ*H*_inh_ = 0 for Co_25_Fe_75_/MAO.

## Discussion

The measured Gilbert damping constant—<1 × 10^−3^—is extremely low for metallic FMs and, remarkably, is comparable to those reported for YIG films^7^. The lowest *α* (7.1 × 10^−4^) for our epitaxial Co_25_Fe_75_ films is considerably lower than the values reported for polycrystalline Co_25_Fe_75_ films^23^ (2.1 × 10^−3^) and epitaxial Fe films (1.9 × 10^−3^)^22^. Considering our measured damping constant also includes contributions from spin pumping into the Cr capping layer^37^ and radiative loss into the environment and stripline^23^, the intrinsic damping of our Co_25_Fe_75_ films should approach the calculated intrinsic value in the mid-low 10^−4^ regime^23–26^.

In summary, we observe extremely low magnetic damping in Co_25_Fe_75_ epitaxial thin films with excellent crystalline quality. This is the first direct measurement of a Gilbert damping constant in the 10^−4^ regime for metallic FM films, making it an ideal material for exploration of spintronic applications requiring metallic FMs.

### Data availability

Data that support the findings of this report are available from the corresponding author upon request.

## Electronic supplementary material

Peer Review File

## References

[1] Sun YY (2012). Growth and ferromagnetic resonance properties of nanometer-thick yttrium iron garnet films. Appl. Phys. Lett..

[2] Sun YY (2013). Damping in yttrium iron garnet nanoscale films capped by platinum. Phys. Rev. Lett..

[3] Serga AA, Chumak AV, Hillebrands B (2010). YIG magnonics. J. Phys. D.

[4] Qu D, Huang SY, Hu J, Wu RQ, Chien CL (2013). Intrinsic spin seebeck effect in Au/YIG. Phys. Rev. Lett..

[5] Lin T, Tang C, Shi J (2013). Induced magneto-transport properties at palladium/yttrium iron garnet interface. Appl. Phys. Lett..

[6] d’Allivy Kelly O (2013). Inverse spin Hall effect in nanometer-thick yttrium iron garnet/Pt system. Appl. Phys. Lett..

[7] Brangham JT (2016). Thickness dependence of spin Hall angle of Au grown on Y_3_Fe_5_O_12_ epitaxial films. Phys. Rev. B.

[8] Wang HL (2013). Large spin pumping from epitaxial Y_3_Fe_5_O_12_ thin films to Pt and W layers. Phys. Rev. B..

[9] Heinrich B (2011). Spin pumping at the magnetic insulator (YIG)/normal metal (Au) interfaces. Phys. Rev. Lett..

[10] Meier D (2015). Longitudinal spin seebeck effect contribution in transverse spin Seebeck effect experiments in Pt/YIG and Pt/NFO. Nat. Commun..

[11] Prakash A, Brangham J, Yang FY, Heremans JP (2016). Spin seebeck effect through antiferromagnetic NiO. Phys. Rev. B.

[12] Costache MV, Sladkov M, Watts SM, van der Wal CH, van Wees BJ (2006). Electrical detection of spin pumping due to the precessing magnetization of a single ferromagnet. Phys. Rev. Lett..

[13] Saitoh E, Ueda M, Miyajima H, Tatara G (2006). Conversion of spin current into charge current at room temperature: inverse spin-Hall effect. Appl. Phys. Lett..

[14] Shikoh E (2013). Spin-pump-induced spin transport in ***p***-type Si at room temperature. Phys. Rev. Lett..

[15] Farle M (1998). Ferromagnetic resonance of ultrathin metallic layers. Rep. Prog. Phys..

[16] Liu X, Zhang W, Carter MJ, Xiao G (2011). Ferromagnetic resonance and damping properties of CoFeB thin films as free layers in MgO-based magnetic tunnel junctions. J. Appl. Phys..

[17] Mosendz O (2010). Detection and quantification of inverse spin hall effect from spin pumping in permalloy/normal metal bilayers. Phys. Rev. B.

[18] Myers EB, Ralph DC, Katine JA, Louie RN, Buhrman RA (1999). Current-induced switching of domains in magnetic multilayer devices. Science.

[19] Liu LQ (2012). Spin-torque switching with the giant spin hall effect of tantalum. Science.

[20] Schreiber F, Pflaum J, Frait Z, Muhge T, Pelzl J (1995). Gilbert damping and G-factor in Fe_*x*_Co_1−*x*_ alloy-films. Solid State Commun..

[21] Scheck C, Cheng L, Bailey WE (2006). Low damping in epitaxial sputtered iron films. Appl. Phys. Lett..

[22] Scheck C, Cheng L, Barsukov I, Frait Z, Bailey WE (2007). Low relaxation rate in epitaxial vanadium-doped ultrathin iron films. Phys. Rev. Lett..

[23] Schoen MAW (2016). Ultra-low magnetic damping of a metallic ferromagnet. Nat. Phys..

[24] Oogane M (2006). Magnetic damping in ferromagnetic thin films. Jpn J. Appl. Phys. Part 1.

[25] Mankovsky S, Kodderitzsch D, Woltersdorf G, Ebert H (2013). First-principles calculation of the Gilbert damping parameter via the linear response formalism with application to magnetic transition metals and alloys. Phys. Rev. B.

[26] Lounis S, Dias MD, Schweflinghaus B (2015). Transverse dynamical magnetic susceptibilities from regular static density functional theory: evaluation of damping and g shifts of spin excitations. Phys. Rev. B.

[27] Hauser AJ (2012). Fully ordered Sr_2_CrReO_6_ epitaxial films: a high temperature ferrimagnetic semiconductor. Phys. Rev. B.

[28] Du CH (2013). Control of magnetocrystalline anisotropy by epitaxial strain in double perovskite Sr_2_FeMoO_6_ films. Phys. Rev. Lett..

[29] Peters B (2013). Epitaxial films of heusler compound Co_2_FeAl_0.5_Si_0.5_ with high crystalline quality grown by off-axis sputtering. Appl. Phys. Lett..

[30] Wang HL, Du CH, Hammel PC, Yang FY (2014). Antiferromagnonic spin transport from Y_3_Fe_5_O_12_ into NiO. Phys. Rev. Lett..

[31] Du CH, Wang HL, Hammel PC, Yang FY (2015). Y_3_Fe_5_O_12_ spin pumping for quantitative understanding of pure spin transport and spin hall effect in a broad range of materials (invited). J. Appl. Phys..

[32] Gallagher JC (2017). Robust zero-field skyrmion formation in FeGe epitaxial thin films. Phys. Rev. Lett..

[33] Kittel, C. *Introduction to Solid State Physics* 7th. edn (Wiley, 1996).

[34] Liu X (2005). Perpendicular magnetization reversal, magnetic anisotropy, multistep spin switching, and domain nucleation and expansion in Ga1-xMnxAs films. J. Appl. Phys..

[35] Taniguchi T, Yakata S, Imamura H, Ando Y (2008). Determination of penetration depth of transverse spin current in ferromagnetic metals by spin pumping. Appl. Phys. Express.

[36] Kalarickal SS (2006). Ferromagnetic resonance linewidth in metallic thin films: comparison of measurement methods. J. Appl. Phys..

[37] Du CH, Wang HL, Yang FY, Hammel PC (2014). Systematic variation of spin-orbit coupling with d-orbital filling: surprisingly large inverse spin hall effect in 3d transition metals. Phys. Rev. B.

